# Perceptions and interest in applied educational neuroscience: a diagnostic study of Brazilian teachers

**DOI:** 10.3389/fpsyg.2026.1895776

**Published:** 2026-08-06

**Authors:** Marcela Prado Siqueira, Analía Arévalo, Guilherme Lepski

**Affiliations:** 1 Department of Psychiatry, Medical School, University of São Paulo, São Paulo, Brazil; 2 Laboratory of Experimental Surgery (LIM26), Medical School, University of São Paulo, São Paulo, Brazil; 3 Department of Neurology, Medical School, University of São Paulo, São Paulo, Brazil

**Keywords:** Brazilian education, early childhood education, educational neuroscience, gender inequalities, higher learning, Institutional differences, teacher training

## Abstract

The integration of educational neuroscience into teacher professional development may support evidence-informed practice, but little is known about Brazilian educators' perceptions of their own knowledge, interests, and their preferred training formats. In this cross-sectional exploratory study, 204 active educators recruited through professional networks and social media completed an online survey. The instrument assessed self-perceived knowledge and interest across ten neuroscience-related topics and preferences for six training formats. Differences in self-perceived knowledge were observed across gender and teaching level; female educators reported greater interest in learning about the Child's brain, and educators in higher levels reported knowing more than educators in other levels regarding Scientific Literacy and Critical Thinking, as well as Neurodevelopment and Brain Plasticity. Training-format preferences also varied across groups. Building on previous work from our group that tested neuroscience knowledge and neuromyth endorsement in over 2,700 Brazilian participants, these new findings describe perceived competence and learning preferences in a group of educators from different areas that may inform the development of targeted training materials.

## 1 Introduction

Teaching in Brazil has been associated with chronic professional devaluation and working conditions that negatively impact the quality of teachers' mental health (Langrafe Junior et al., 2025; Oliveira et al., 2016). Also, there are clear contrasts among Brazilian educational networks. While teachers in private schools often report greater satisfaction with compensation and infrastructure, those teaching in the public schools report significant dissatisfaction and skepticism regarding career plans and public policies (Lemos and Fernandes, 2022).

The devaluation of the profession is a central problem, manifesting both in negative social perception and low remuneration, and is cited as one of the main reasons for leaving the career. Data collected in 2024 by SEMESP (*Sindicato das Entidades Mantenedoras de Estabelecimentos de Ensino Superior no Estado de São Paulo*), the institute that currently represents higher education providers in Brazil, indicated that approximately 79.4% of interviewed teachers have already considered abandoning teaching, and have felt discouraged and frustrated with the future. This feeling is aggravated by excessive working hours and the need for many professionals to seek additional sources of income to maintain a decent standard of living.

Another alarming challenge reported by SEMESP is the increase in violence within the school environment, with more than half of interviewed teachers (52%) reporting they have already suffered some type of aggression, predominantly verbal, and coming from the students themselves. Additionally, educators have noticed negative changes in student behavior in recent years, highlighting a lack of interest, increased indiscipline, and the excessive use of technology as barriers to learning. In social representations of public schools, a process of mutual blaming emerges, pointing to a lack of family commitment and government negligence in the management and investment of resources (SEMESP, 2024).

In a laudable attempt to understand their students' behavior and improve their teaching, some educators will often look for information regarding neuroscience and its application to education (Torrijos-Muelas et al., 2021). Here is where a critical problem emerges: often, teachers' searches overwhelmingly lead them to persistent “neuromyths,” which are false conceptions or misguided simplifications about brain function applied to education. Research indicates that a significant portion of Brazilian teachers, both in training and in practice, believe in ideas based in pseudoscience, such as the existence of “learning styles” (students learn better if taught only through their preferred modality —visual, auditory, or kinesthetic—VAK), the myth that we use only 10% of our brain, and the idea that hemispheric dominance (left vs. right brain) explains individual differences (Torrijos-Muelas et al., 2021; Dekker et al., 2012).

These false beliefs are problematic because they waste time, effort, and financial resources on ineffective pedagogical practices, and they can potentially undermine teachers' confidence in science when such methods fail. Curiously, studies show that having a genuine interest in neuroscience or reading popular science magazines does not necessarily protect teachers from these myths; often, high general knowledge about the brain correlates with a greater acceptance of neuromyths, as professionals find it difficult to distinguish scientific facts from marketed pseudoscience (Simoes et al., 2022; Dekker et al., 2012). Furthermore, the allure of explanations that use brain images can lead to the acceptance of irrelevant or erroneous theories in the school environment (Torrijos-Muelas et al., 2021).

Simoes et al. (2022) surveyed Brazilian educators' knowledge about neuroscience and education and reported significant regional disparities, with educators in Brazil's southern region demonstrating higher neuroscience literacy compared with educators in the North and Northeast. This geographical variation suggests that access to scientific information and continuing education programs is not uniform across the national territory, which can directly influence the quality of pedagogical practices in different states. Also, professionals teaching higher educational levels demonstrated more robust general knowledge about the brain than those teaching lower levels. One positive observation was that teachers with more than 40 years of teaching experience obtained higher scores, suggesting that in some settings, pedagogical practice and professional longevity contribute to the ongoing improvement of an educator's scientific knowledge base.

In Chile, a 1-year training course showed positive results in improving teachers' neuroscience literacy, as well as reducing the belief in neuromyths (Ferreira and Rodríguez, 2022). However, simple passive exposure to content may not be enough. Many have argued that it is more efficient to directly confront myths with scientific evidence through lectures or refutation texts (Adiguzel et al., 2025; Lithander et al., 2024; Torrijos-Muelas et al., 2021; Dekker et al., 2012). Therefore, it is possible to conclude that advanced schooling alone does not guarantee immunity to erroneous concepts; there seems to be a clear need for university teaching degree curricula and graduate and extension programs to incorporate solidified knowledge about learning and the brain, and actively promote the training of critical thinking skills.

The ultimate goal, while seemingly elusive, is to establish a more fluid communication between researchers who study how to apply neuroscientific findings in the classroom and educators who are the drivers and implementors of such findings. But with all the problems faced by teachers today, as cited above, how can we build and maintain this bridge? As discussed, having an interest in the biological underpinnings of learning is the first necessary step, but we still lack information on what the best way of offering training is, especially for educators working in diverse contexts. To address these challenges, we used a targeted survey to investigate three fundamental questions: (I) what do Brazilian teachers claim to know about neuroscience?; (II) what specific topics within the field of neuroscience and education are they most interested in learning?; and (III) what are their preferred formats for receiving this information in professional development settings? Our greater goal was to discover answers that can better inform the design of more effective and targeted teacher training programs.

## 2 Material and methods

### 2.1 Participants

This cross-sectional study included a convenience sample of 204 Brazilian educators who were contacted via professional educational networks and social media platforms. Both the first author and co-authors of our previous publications work in education, which facilitated our network reach. We only included currently active teachers.

The study's final sample consisted of *n* = 204 educators. Regarding age distribution, participants were categorized into four main groups: 18–21 years (*n* = 18), 22–35 years (*n* = 59), 36–50 years (*n* = 89), and over 50 years (*n* = 35); three participants did not report their age. The sample was predominantly female (*n* = 164), with 40 male participants. In terms of professional experience, most were seasoned educators, with 118 participants having taught for over 10 years, followed by 45 participants with 6–10 years of experience, and 37 participants with 1–5 years; four individuals did not provide this information. Regarding institutional affiliation, 97 educators worked in private schools, 88 in public schools, and 19 reported working in both types of institutions.

Participants also represented various educational levels: Early Childhood Education (ECE, *n* = 39), Elementary School (ES, *n* = 47), Middle School (MS, *n* = 25), High School (HS, *n* = 51), Higher Education (HE, *n* = 36), and Education for Young Adults (EYA, *n* = 6). Because some taught different levels, we grouped them according to the highest level taught. Finally, the breakdown of areas of expertise revealed that 21 educators had a specialty in Biological Sciences, 15 in Health Sciences, and 13 identified with both Biological and Health Sciences, while most (*n* = 155) were from other areas of knowledge (Humanities, Languages and Mathematics).

### 2.2 Survey instrument and procedures

Data were collected using an online survey hosted on Google Forms, which was made available online between March 16^th^, 2024, and August 23^rd^, 2025. The English version of the questionnaire is provided in Supplementary material.

The survey was divided into three main sections. The first section gathered socio-demographic and professional profiles, including age, gender, teaching experience (years), type of institution (public or private), highest level of teaching, and area of expertise. Additionally, participants were asked about their perception of neuroscience's contribution to teaching and their interest in professional development in this field.

The second section assessed self-perceived knowledge and interest across 10 areas of knowledge: 1. ADHD (Attention Deficit Hyperactivity Disorder), 2. ASD (Autistic Spectrum Disorder), 3. Memory and Learning, 4. Learning Disorders, 5. The Child's Brain, 6. The Adolescent Brain, 7. Dyslexia, Dysgraphia and Dyscalculia, 8. Neurodevelopment and Brain Plasticity, 9. Emotions and Social-Emotional Learning, and 10. Scientific Literacy and Critical Thinking. For each theme, educators rated two aspects: (i) their self-reported level of knowledge and (ii) the perceived relevance and interest in the topic, both measured on a 6-point Likert scale (0 = no preference to 5 = strong preference).

The final section evaluated teachers' preference for the format in which information should be delivered: 1. asynchronous recorded classes, 2. synchronous online classes, 3. in-person classes, 4. individual reading assignments, 5. synchronous workshops, and 6. in-person workshops. These preferences were also measured on a scale of 0 to 5 (0 = no preference to 5 = strong preference).

The questionnaire was developed for this diagnostic study and was not subjected to a formal pre-study psychometric validation process. Its design was based on educator feedback obtained by the first author (an educator with more than 13 years of experience in both public and private Brazilian schools), as well as co-authors of our previous study (Simoes et al., 2022), in which we surveyed more than 1,600 educators on neuroscience knowledge and neuromyth endorsement. Additionally, internal-consistency estimates were calculated *post hoc* for the three multi-item sections which yielded a Cronbach's alpha = 0.934 for the 10 self-perceived knowledge items, alpha = 0.950 for the 10 interest/relevance items, and alpha = 0.645 for the six training-format preference items. The first two sections therefore showed excellent internal consistency, whereas the format-preference section showed moderate consistency.

The study was carried out in accordance with the Declaration of Helsinki and approved by the ethics committee of the Medical School of the University of Sao Paulo (protocol number CAAE 76331623.9.0000.0068). The form explained the study's purpose, indicated that anonymity would be protected at all times by never collecting (or storing) names or any other identifying information and coding answers so that these could not be associated with a particular participant. The first page of the survey (the consent form) explained these issues and asked participants whether they agreed with their anonymous answers being used in the research study. Only after providing consent, could participants access the remainder of the survey.

### 2.3 Quantitative analysis

At the beginning of the survey, when educators responded about the relevance of neuroscientific knowledge applied to education, an almost unanimous consensus was observed, although this estimate may be influenced by convenience sampling and self-selection. Out of the 204 participants, only one individual did not consider neuroscience knowledge to be relevant to educational practice. There was also a strong desire for further professional development, with 186 (91%) participants indicating an interest in pursuing more information in the area, and only 18 educators reporting no such interest.

To assess the distribution of the collected data, a Shapiro-Wilk test of normality was conducted, which is most powerful and sensitive, especially for smaller sample sizes. This test indicated that the scores did not follow a normal distribution (*p* < 0.05). Thus, for all subsequent comparisons and correlations, Wilcoxon and Kruskal-Wallis non-parametric tests were applied. In addition, Holm's sequential correction within each grouping family were conducted, which controls the family-wise error rate while being less conservative than Bonferroni. For teaching level and area of expertise, the Kruskal-Wallis omnibus tests were corrected first; pairwise tests were interpreted only for outcomes surviving the corrected omnibus analysis, and those pairwise *p* values were then Holm-adjusted. All analyses were conducted using JMP Student Edition 19.0 (SAS Institute, Cary, NC, USA).

Three participants had missing age data. The survey outcome variables, gender, years teaching, institution type, highest teaching level, area indicators, relevance, and interest had no missing observations. Descriptive statistics for age used available cases (*n* = 201). Inferential analyses were complete-case analyses for the variables included in each test. Because outcome data were complete, all 204 participants contributed to the outcome analyses. Also, because there were 18 participants who reported working in both private and public institutions, the private-versus-public comparison excluded these, yielding *n* = 186. See Supplementary Table 1 for all Descriptive statistics for all Likert outcomes.

## 3 Results

### 3.1 Gender

**Figure 1:**
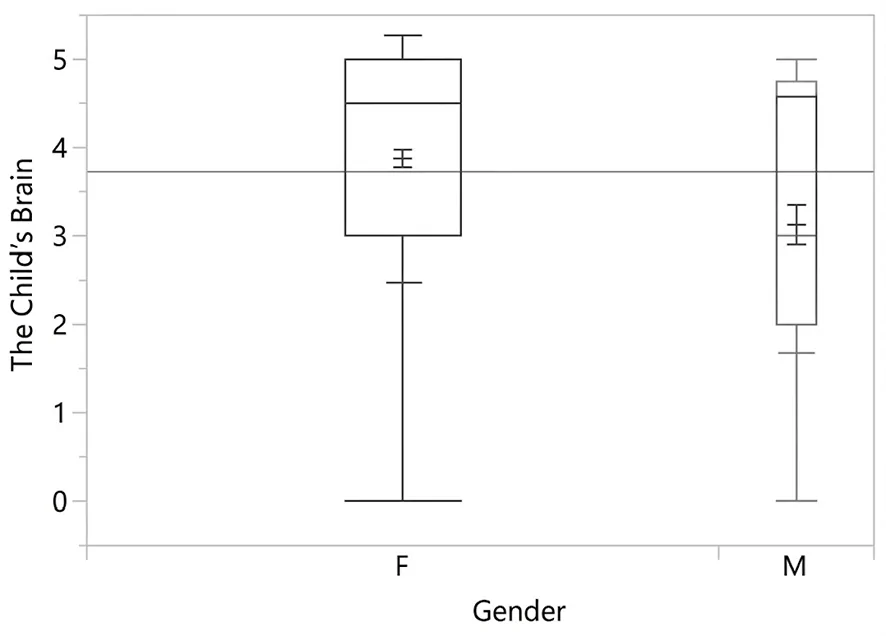
The topic of the Child's brain was rated significantly more important by women than men. F: Female; M: Male.

When we analyzed gender collapsed across all other categories, only one gender-related outcome remained significant: female educators reported greater interest in the topic of the Child's Brain than male educators (*p* < 0.0015; see Figure 1 and Table 1).

**Table 1:** Multiplicity-corrected principal analyses.

Family/outcome	Comparison	Raw *p*	Holm *p*	Effect	Group 1 median	Group 2 median	Interpretation
Gender/interest in child's brain	Female vs. male	0.0015	0.0393	*R* = 0.222	3.0	3.9	Significant; small effect
Institution type/26 outcomes	Private vs. public	–	all >0.05	–	–	–	No comparison survived Holm correction
Teaching level/scientific literacy/critical thinking	Six-level Kruskal-Wallis	0.000000	0.000003	epsilon^2^ = 0.178	–	–	Significant omnibus effect
Teaching level/neurodevelopment/ brain plasticity	Six-level Kruskal-Wallis	0.000882	0.022057	epsilon^2^ = 0.080	–	–	Significant omnibus effect
Area of expertise/26 outcomes	Four groups	–	all >0.05	–	–	–	No omnibus outcome survived Holm correction

### 3.2 Type of institution

Public and private school teachers did not differ on how they rated the relevance of neuroscience to their field or their overall interest in neuroscience. However, distinct patterns emerged concerning preferences for information delivery. While educators from public schools tended to prefer Asynchronous Recorded Classes, private school teachers scored slightly higher, comparatively, on all other formats; however, none of these comparisons reached significance.

### 3.3 Highest level of teaching

**Figure 2:**
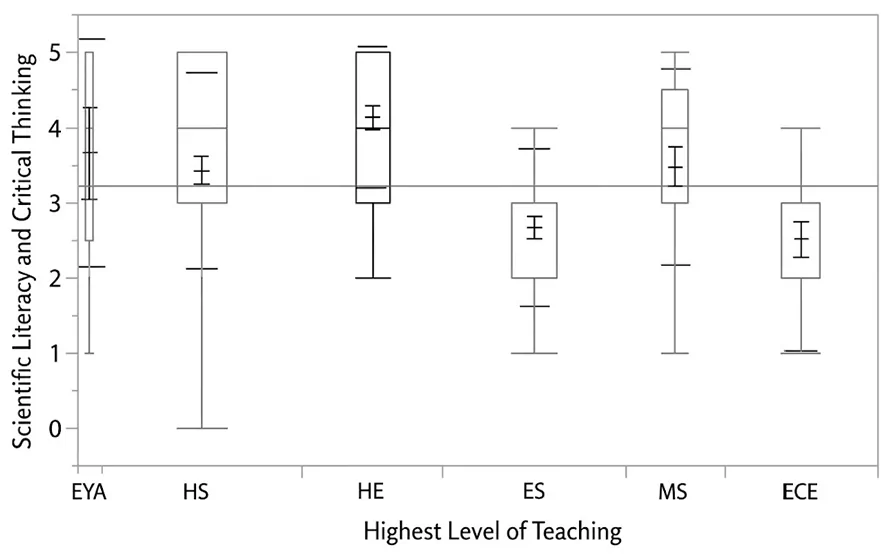
Higher education educators ranked their knowledge in scientific literacy and critical thinking higher than ECE and ES teachers. EYA, education of young-adults and adults; HS, high school; HE, higher education; ES, elementary school; MS, middle school; ECE, early childhood education.

Because many teachers taught students at several levels, we grouped them based on their highest level of teaching. Following Holm correction, teaching level remained associated with self-perceived knowledge of Scientific Literacy and Critical Thinking (Holm-adjusted omnibus *p* = 3.36 × 10^−6^; epsilon^2^ = 0.178) and Neurodevelopment and Brain Plasticity (Holm-adjusted omnibus *p* = 0.022; epsilon^2^ = 0.080). Holm-adjusted pairwise comparisons indicated that Higher Education teachers reported higher Scientific Literacy and Critical Thinking ratings than Early Childhood Education and Elementary School teachers (see Figure 2), and higher Neurodevelopment and Brain Plasticity ratings than Early Childhood Education, Elementary School, and High School teachers. No area-of-expertise omnibus Comparison remained significant after correction (see Table 1). See Table 2 for all significant Holm Adjusted teaching-level comparisons.

**Table 2:** Significant Holm-adjusted teaching-level pairwise comparisons.

Outcome	Comparison	Raw *p*	Holm *p*	*r*	Median 1	Median 2
Scientific literacy/critical thinking	Early childhood education vs. high school	0.002697	0.032361	0.317	2.0	4.0
Scientific literacy/critical thinking	Early childhood education vs. higher education	0.000002	0.000027	0.550	2.0	4.0
Scientific literacy/critical thinking	Elementary school vs. middle school	0.002972	0.032693	0.351	3.0	4.0
Scientific literacy/critical thinking	Elementary school vs. high school	0.001850	0.024055	0.315	3.0	4.0
Scientific literacy/critical thinking	Elementary school vs. higher education	0.000000	0.000001	0.600	3.0	4.0
Neurodevelopment/brain plasticity	Early childhood education vs. higher Education	0.000134	0.002015	0.442	2.0	3.5
Neurodevelopment/brain plasticity	Elementary school vs. higher education	0.001378	0.019286	0.352	3.0	3.5
Neurodevelopment/brain plasticity	High school vs. higher education	0.001670	0.021713	0.337	2.0	3.5

### 3.4 Area of expertise

Next, we compared educators with a background in Health and Biological Sciences with those from other areas of expertise, such as Humanities, Languages, and Mathematics. While the original analyses showed a tendency for teachers with Health Sciences expertise to report higher knowledge than other teachers in the areas of Memory and Learning, Neurodevelopment and Brain Plasticity, and Scientific Literacy and Critical Thinking, none of these comparisons survived the Holm correction, suggesting teacher specialty training may not prepare teachers better for the type of knowledge tested in our survey.

Concerning perceived relevance and interest in further knowledge, teachers in the Health Sciences and those in both Health and Biological Sciences scored slightly higher than those from other areas, though no statistical significance was found. Likewise, information delivery preferences did not differ significantly among the various areas of expertise.

## 4 Discussion

The primary objective of this study was to map neuroscience literacy, interests, and training preferences among Brazilian educators, connecting these data to the historical and social reality of our school system. By understanding what teachers know and what they want to learn, this research aims to identify the real challenges in bringing neuroscience into the classroom. To address these challenges, our survey asked three fundamental questions: (I) what do Brazilian teachers claim to know about neuroscience?; (II) what specific topics within the field of neuroscience and education are they most interested in learning?; and (III) what are their preferred formats for receiving this information in professional development settings? Our greater goal was to discover answers that can better inform the design of more effective and targeted teacher training programs.

Based on previous findings from our own group and others' (Simoes et al., 2022), we predicted that educators in private schools, as well as those teaching higher levels, would report greater knowledge in certain neuroscience topics. While a first set of analyses revealed some differences in self-reported knowledge between female versus male educators and educators teaching in private versus public schools, most differences did not survive statistical correction. Female educators did report significantly higher interest in the topic of the Child's Brain. Also, in line with our predictions, those teaching in higher education reported significantly higher self-perceived knowledge in the topics of Scientific Literacy and Critical Thinking and Neurodevelopment and Brain Plasticity.

In the next sections, we discuss the implications of these findings in light of historical data and previous findings.

### 4.1 Gender inequalities in historical and professional pathways

Regarding differences between genders, female educators reported attributed significantly greater importance to the topic of “The Child's Brain,” and in an uncorrected analysis, male educators reported higher levels of knowledge in “Scientific Literacy and Critical Thinking.”

The finding that women reported greater knowledge and desire to study The Child's Brain may be analyzed through two lines of thinking: the *Scientification of Care* and *Professional and Personal Identity*.

#### 4.1.1 Scientification of care

Since the 1800s, female teachers have been socially expected to possess “maternal qualities” and a natural inclination toward caring for and understanding children (Lima, 2020). However, as female educators are transitioning from “vocation-based” teaching to a more “professionalized” science-based approach, they may be more inclined to use neuroscience to reinforce their understanding of early childhood education, a level in which they have historically been the primary practitioners.

#### 4.1.2 Professional and personal identity

For many female educators, expertise in child development and literacy is not just a technical skill but a core component of their professional identity; historically, within a society that offered few professional pathways to women, teaching also represented the closest option to achieving financial and intellectual freedom as an individual (Almeida, 1998, 2006).

Importantly, gender differences in education may be driven uneven representation across educational sectors. In the current study, 70% of men work in private schools and 65% teach at high educational levels (27.5% in Higher Education and 37.5% in High School), whereas a mere 2.5% teach in Early Childhood Education. This distribution corroborates a recent literature review by Dourado and Moreira (2026) addressing gender differences in teaching highlighting that issues of remuneration and low social prestige remain highly pervasive. The fact that female teachers have been historically treated as “childcare providers” or merely as service providers, occupying devalued positions perceived not to require extensive training, has further contributed to lower pay for women (Dourado and Moreira, 2026; Rosemberg, 1997). Thus, men predominantly occupy higher-paying educational stages as well as school management and coordination positions (Dourado and Moreira, 2026), making it more difficult for women to invest in their professional development. This financial vulnerability is further aggravated in under-resourced school environments, where female teachers often use their own financial means to guarantee teaching goals, ultimately reproducing the historical model of the “dedicated and altruistic teacher”^1^ (Carvalho et al., 2018; p. 15).

### 4.2 Institutional contexts and their impact on teachers' professional development opportunities

Most studies conducted in Brazil to date have shown better working conditions for teachers in private schools (Langrafe Junior et al., 2025). In Simoes et al. (2022), private school teachers scored better on recognizing neuromyths relative to those in public schools.

While we predicted that private school teachers would report higher self-perceived knowledge, statistical correction eliminated these initial findings in the current data. According to Oliveira et al. (2016), private schools have greater flexibility to develop their own personnel management policies, which promotes higher job satisfaction among teachers. Conversely, data show that the highest rates of medical leave due to mental health issues, such as burnout, depression, and anxiety, occur within the public school system (Langrafe Junior et al., 2025; Oliveira et al., 2016). In contrast, public school teachers, despite holding graduate degrees, do not receive the same incentives from the public agencies that manage their institutions. Furthermore, they lack encouragement to apply the competencies they already possess, which could be fostered through salary increases or better working conditions (Oliveira et al., 2016; Sobrinho et al., 2023).

Perhaps the most interesting finding was that educators in private and public schools did not differ significantly from each other when asked about what they would like to know regarding applied educational neuroscience topics. Thus, while most would agree that these different contexts provide educators with unequal advantages in terms of training, preparedness and overall knowledge, their desires and interests are quite similar, and this should be considered when designing educational materials to meet these needs. Interestingly, while these also did not reach significance, some variations emerged regarding their preferred formats for receiving information. Teachers working in public schools tended to prefer Asynchronous Recorded Classes; because this format allows for greater flexibility, it may be particularly attractive for educators with a heavy workload (both in the classroom and in planning), as well as those who receive low pay, yet another obstacle to investing in one's professional development (Lemos and Fernandes, 2022). On the other hand, private school teachers tend to prefer all other formats, with a stronger emphasis on in-person learning; greater financial and institutional incentives for this type of format likely influence private teachers' preference for this method. According to Lemos and Fernandes (2022), because of the outsourcing of the pedagogical process in some for-profit private schools, these institutions adopt educational systems that provide products and services (e.g., continuing education during the workday), ensuring better use of teaching time without generating an administrative overload.

An interesting point that can also be observed is the gender breakdown in the faculty composition across different institutions. Several studies show that in public schools, the teaching staff is predominantly composed of female teachers, which perpetuates low remuneration for women, as discussed in the previous section, in addition to the mental and emotional overload that female teachers have historically carried (Dourado and Moreira, 2026; Langrafe Junior et al., 2025).

### 4.3 From early childhood to higher education: disparities in teachers' (neuro)scientific knowledge

Initial teacher training in Brazil, governed by Resolution CNE/CP No. 4, from May 29, 2024, presents all the criteria that must be followed by higher education institutions offering teaching degree programs (*licenciaturas*) across different areas of knowledge. This Resolution does not contain specific neuroscience-related content; however, contents related to the science of learning are present in the chapters dealing with the foundations and principles of teacher training (Chapter II, Art. 4, Sole Paragraph), the common base and the profile of graduates from initial training (Chapter III, Art. 10, items XVI and XVII), and the structure and curriculum of degree programs (Chapter IV, Art. 13, Item I - d). At this point, it is worth noting that to practice the teaching profession in basic education in Brazil, professionals must hold at least a higher education degree, which in this case is the *licenciatura*.

Even with all these specifications, a gap remains evident in initial training, particularly among licensed teachers in Early Childhood Education (ECE) and Elementary School (ES). In the study by Simoes et al. (2022), educators most likely to endorse neuromyths were precisely those teaching lower grades. This is troubling given that knowledge about early development (typical and atypical) is particularly critical for ES and ECE teachers, who are precisely in the right place to offer insights and recommend potential interventions. Similarly, in the present study, ES and ECE teachers (but especially those in ECE), reported lower self-perceived knowledge regarding all the surveyed topics.

The major discrepancy that emerged in our study was between the two extremes of educational levels: Early Childhood Education (ECE) teachers and Higher Education (HE) professors. In Brazil, to teach in higher education, professors must hold, at minimum, a graduate-level specialization course or a master's degree. Thus, these instructors possess, at the very least, an additional level of formal education compared with those teaching at the opposite extreme.

Looking more closely at the data, the topics for which self-perceived knowledge differed most between ECE and HE educators were: Neurodevelopment and Brain Plasticity, and Reading Scientific Articles and Critical Literacy. Among these themes, one of the most alarming is Neurodevelopment and Brain Plasticity, a topic critically relevant for ECE teachers. Brazilian studies, such as those by Silva and Gama (2022) and Machado (2025), highlight how much these teachers could benefit from neuroscientific knowledge in their teaching practice, and emphasize the importance of including neuroscience-based courses in degree programs nationwide, something that currently is blatantly lacking (Grossi et al., 2024).

Another interesting point to address relates to the topic of Reading Scientific Articles and Critical Literacy. As previously mentioned, in order to teach at their level, HE professors must trace a longer academic trajectory, which may explain the discrepancy in findings between them and ECE and ES teachers. However, according to the Resolution governing initial teacher training in Brazil (BRASIL, 2024b), degree programs must train critical thinking and problem-solving skills (Chapter III, Art. 7, Item IV—a), in addition to collaborating with scientific research in the field of education whenever possible, allowing them to constantly reflect upon and apply scientific knowledge to their teaching practice (Chapter III, Art. 10, Item XX). Furthermore, it is important to mention that developing proficiency in scientific reading is not an outcome of general higher education; rather, it requires intentional training and extensive practice to achieve scientific literacy. Otherwise, empirical data remains a significant barrier for most educators.

### 4.4 How areas of expertise shape (neuro)scientific knowledge

To categorize the areas of expertise included in our questionnaire, we used the classification system of the Coordination for the Improvement of Higher Education Personnel (*Coordenação de Aperfeiçoamento de Pessoal de N*í*vel Superior*—CAPES), a foundation under the Brazilian Ministry of Education (MEC) (BRASIL, 2024a). Accordingly, the division of fields comprised Linguistics, Letters, and Arts (Languages); Exact and Earth Sciences and Engineering (Mathematics); Human Sciences and Applied Social Sciences (Humanities); Biological Sciences; and Health Sciences. Thus, we also conducted a comparative analysis between teachers in the fields of Biological and Health Sciences (due to the closer proximity of their background to neurosciences) and teachers from all other areas.

While a previous study from our group observed differences in neuroscience knowledge and neuromyth endorsement among laypeople from different fields of study (Arévalo et al., 2022), with those from the biological sciences responding best and those in the health sciences responding surprisingly less so, in the current study, the area of expertise comparison did not predict self-perceived knowledge after statistical corrections.

## 5 Limitations

This study has a few important limitations to be addressed in future research. First, we collected self-reported responses rather than an objective and direct assessment of neuroscientific knowledge. The questionnaire was not subjected to a formal pre-study psychometric validation process; although *post-hoc* internal-consistency estimates were favorable for the knowledge and interest domains, they do not establish content, construct, or criterion validity. Second, classifying teachers who worked at multiple levels according to the highest level taught may have obscured part of their professional experience, and multiple comparisons through Holm correction reduced the number of statistically supported group differences. Third, we did not address regional differences in our survey. Brazil is a country of continental proportions and future work should target different regions to assess differences among them. Future research should thus encompass larger and multi-regional samples of educators.

## 6 Conclusion

In this convenience sample of Brazilian educators, interest in educational neuroscience was high, while self-perceived knowledge and preferred training formats varied across some participant groups. After correction for multiple testing, the most robust differences concerned teaching level, particularly self-perceived familiarity with Scientific Literacy and Critical Thinking and with Neurodevelopment and Brain Plasticity. These findings do not demonstrate differences in objective neuroscience competence and do not permit conclusions about neuromyth endorsement or causal social mechanisms. They provide exploratory information that may inform the design of future teacher-training studies using validated instruments, objective knowledge measures, probability-based or broader multi-regional sampling, and direct evaluation of educational outcomes.

Based on these findings, future developments will focus on the creation of a curriculum proposal designed to mitigate the identified gaps in neuroscientific knowledge among Brazilian educators. This proposal aims to support teachers who wish to learn about and discuss educational neuroscience, directly connecting these insights to the planning of their lessons and school projects. Grounded in the reading and critical discussion of scientific articles, the training framework would be structured to foster critical thinking regarding the diverse pedagogical challenges that arise in daily classroom routines. We will also adapt the material to several formats to test whether applying these in different contexts can initially facilitate teachers' access to information. Ultimately, this initiative intends to yield two primary outcomes: the publication of a foundational pedagogical model aimed at translating neuroscientific research into education, alongside the implementation of practical training modules in various formats for teacher professional development courses.

## Data Availability

The original contributions presented in the study are included in the article/Supplementary material, further inquiries can be directed to the corresponding author.

## Footnotes

1 Translated by the authors from Portuguese.

## References

[B1] Adiguzel O. C. Potvin P. Sarrasin J. B. Vanhoolandt C. Corfdir A. Japashov N. . (2025). Belief in neuromyths among primary school teachers: a cross-national study of 11 countries. Trends Neurosci. Educ. 40, 100264. doi: 10.1016/j.tine.2025.100264 40889831

[B2] Almeida J. S. (1998). Mulher e Educação: a Paixão pelo Possí*vel*. São Paulo: UNESP.

[B3] Almeida J. S. (2006). “Vestígios para uma reinterpretação do magistério feminino em Portugal e no Brasil a partir do século XIX,” in O legado educacional do século XIX. 2. eds. S. P. Campinas and D. Saviani (Campinas: Autores Associados), 133–197.

[B4] Arévalo A. Simoes E. Petinati F. Lepski G. (2022). What does the general public know (or not) about neuroscience? effects of age, region and profession in Brazil. Front. Hum. Neurosci. 16, 798967. doi: 10.3389/fnhum.2022.798967 35308611 PMC8930840

[B5] BRASIL (2024a). Coordenação, de Aperfeiçoamento de Pessoal de Ní*vel Superior(CAPES), Tabela de áreas de conhecimento/avaliação. Bras*í*lia*, DF: CAPES. Available online at: https://www.gov.br/capes/pt-br/acesso-a-informacao/acoes-e-programas/avaliacao/instrumentos/documentos-de-apoio/tabela-de-areas-de-conhecimento-avaliacao (Accessed May 18, 2026).

[B6] BRASIL (2024b). Resolução CNE/CP n° *4, de 29 de maio de 2024. Dispõe sobre as Diretrizes Curriculares Nacionais para a Formação Inicial em N*í*vel Superior de Profissionais do Magistério da Educação Escolar Básica*. Diário Oficial da União: seção 1, Brasília, DF, n. 104, p. 26, 03 jun. 2024b. Available online at: https://www.in.gov.br/web/dou/-/resolucao-cne/cp-n-4-de-29-de-maio-de-2024-563084558 (Accessed April 10, 2026).

[B7] Carvalho M. P. d. e. Toledo C. T. Oliveira I. G. d. e. Modesto E. Neto C. d. a. S. (2018). Cuidado e Gerencialismo: para onde vai o trabalho das professoras. educação em Revista. Belo Horizonte34:e203244. doi: 10.1590/0102-4698203244

[B8] Dekker S. Lee N. C. Howard-Jones P. Jolles J. (2012). Neuromyths in education: prevalence and predictors of misconceptions among teachers. Front. Psychol. 3:429. doi: 10.3389/fpsyg.2012.00429 23087664 PMC3475349

[B9] Dourado C. G. V. B. Moreira G. E. (2026). Desigualdade de gênero e desvalorização da profissão docente: aspectos revelados em uma revisão sistemática. Revista Cadernos Cajuína 6:e2898. doi: 10.52641/cadcajv11i6.2898

[B10] Ferreira R. A. Rodríguez C. (2022). Effect of a science of learning course on beliefs in neuromyths and neuroscience literacy. Brain. Sci. 12:811. doi: 10.3390/brainsci12070811 35884619 PMC9312647

[B11] Grossi M. G. R. Oliveira E. S. Fonseca R. G. P. (2024). Currículo, neurociência e a formação de professores. Revista e-Curriculum 22, 1–26. doi: 10.23925/1809-3876.2024v22e59967

[B12] Langrafe Junior A. Maran Z. M. B. Nishiyama A. A. (2025). mentalidade de professores sobre o estresse: estratégias para lidar com os desafios da educação pública. Rev. Bras. Educ. 30:e300112. doi: 10.1590/s1413-24782025300112

[B13] Lemos E. F. U. Fernandes J. S. G. (2022). Escolas pública e particular: representações sociais de professores. Rev. Bras. Educ. 27. doi: 10.1590/s1413-24782022270111

[B14] Lima M. C. A. (2020). Feminização do magistério: o lugar da mulher como professora no triângulo mineiro e alto paranaíba. Rev. Diálogo Educ. 67, 1706–1732. doi: 10.7213/1981-416X.20.067.DS10

[B15] Lithander M. P. G. Geraci L. Karaca M. Hunsberger R. (2024). The effect of correcting neuromyths on students' and teachers' later reasoning. J. Intell. 12:98. doi: 10.3390/jintelligence12100098 39452515 PMC11508907

[B16] Machado M. M. (2025). Neurociência na educação infantil e anos iniciais do ensino fundamental. Rev. Ibero-Am. Humanid. Cienc. Educ. 1:8. doi: 10.51891/rease.v11i8.20677

[B17] Oliveira T. F. Lins V. L. Silva R. M. Fontoura L. V. (2016). Qualidade de vida no trabalho: um estudo comparativo entre professores de escola pública e privada. Psico. Argum. 34, 104–109. Available online at: https://periodicos.pucpr.br/psicologiaargumento/article/view/23311/22431 (Accessed May 16, 2026).

[B18] Rosemberg F. (1997). Educação infantil, educar e cuidar e a atuação profissional. Infanc. Ciranda Educ. 3, 21–26.

[B19] SEMESP (2024). Pesquisa: Perfil e Desafios dos Professores da Educação Básica no Brasil. São Paulo. Available online at: https://www.semesp.org.br/wp-content/uploads/2024/04/resultados-pesqdocenteseb.pdf (Accessed November 27, 2025).

[B20] Silva A. F. d. a. Gama M. L. S. (2022). Neurociência e educação: o desenvolvimento humano da criança na educação infantil. Anais VIII CONEDU. Campina Grande: Realize Editora. Available online at: https://editorarealize.com.br/artigo/visualizar/88481 (Accessed May 17, 2026).

[B21] Simoes E. Foz A. Petinati F. Marques A. Sato J. Lepski G. . (2022). Neuroscience knowledge and endorsement of neuromyths among educators: what is the scenario in brazil? Brain Sci. 12:734. doi: 10.3390/brainsci12060734 35741619 PMC9221520

[B22] Sobrinho A. I. Sousa G. B. Lima J. R. Nunes G. O. (2023). Escolas pública e privada: desigualdades latentes; soluções evidentes. Anais IX CONEDU. Campina Grande: Realize Editora. Available online at: https://editorarealize.com.br/artigo/visualizar/99765 (accessed May 16, 2026).

[B23] Torrijos-Muelas M. González-Víllora S. Bodoque-Osma A. R. (2021). The persistence of neuromyths in the educational settings: a systematic review. Front. Psychol. 11:591923. doi: 10.3389/fpsyg.2020.591923 33510675 PMC7835631

