# Supplementary material for: Perceptions and interest in applied educational neuroscience: a diagnostic study of Brazilian teachers
Source: Front Psychol. 2026 Aug 6;17:1895776. doi: 10.3389/fpsyg.2026.1895776 (PMC13489923; doi:10.3389/fpsyg.2026.1895776)
Supplement: Supplementary file 1 [file Data_Sheet_1.pdf]

Questionnaire items translated from Brazilian Portuguese

**Section 1 - Socio-demographic and professional profiles**
How old are you (in years)?
What is your gender?<br>*Options: Female*<br>*Male*<br>*Prefer not to say*
How many completed years of teaching experience do you have?
In what type of institution do you teach? (Multiple choices allowed)<br>*Options: Private*<br>*Public*
Which educational level(s) do you teach? (Multiple choices allowed)<br>*Options: Early Childhood Education*<br>*Elementary School*<br>*Middle School*<br>*High School*<br>*Education of Young-adults and Adults*<br>*Higher Education*
What is/are the field(s) of knowledge of the subject(s) you teach? (Multiple choices allowed)<br>*Options: Linguistics, Literature, and Arts*<br>*Exact and Earth Sciences*<br>*Human Sciences*<br>*Biological Sciences*<br>*Health Sciences*<br>*Applied Social Sciences*<br>*Engineering*
Do you believe neuroscience knowledge can contribute to your teaching practice?<br>*Options: Yes*<br>*No*
Are you interested in participating in a neuroscience training program for educators?<br>*Options: Yes*<br>*No*
**Section 2 – Self-reported level of knowledge**

Questionnaire items translated from Brazilian Portuguese

Command: Consider the topics below and rate your level of knowledge on a scale from 0 to 5, where 0 means 'not at all' and 5 means 'a lot'.
Memory and Learning
The Child's Brain
ASD (Autistic Spectrum Disorder)
Scientific Literacy and Critical Thinking
Dyslexia, Dysgraphia, and Dyscalculia
Neurodevelopment and Brain Plasticity
ADHD (Attention Deficit Hyperactivity Disorder)
Learning Disorders
Emotions and Social-Emotional Learning
The Adolescent Brain
**Section 3 – Perceived relevance and interest in the topic** Command: Consider the topics below and use the scale from 0 to 5 to rate your level of interest and how relevant they are to you, where 0 means 'none' and 5 means 'high'.
ASD (Autistic Spectrum Disorder)
ADHD (Attention Deficit Hyperactivity Disorder)
Dyslexia, Dysgraphia, and Dyscalculia
Learning Disorders
Memory and Learning
Emotions and Social-Emotional Learning
The Child's Brain
The Adolescent Brain
Scientific Literacy and Critical Thinking
**Section 4 – Preference for the format for information delivery** Command: Rate your preferred formats for receiving/studying information on a scale of 0 to 5, in which 0 means no preference and 5 means strong preference.
Asynchronous/ recorded classes
Synchronous online classes
In-person classes
Individual reading assignments
Synchronous online workshops
In-person workshops
