# Supplementary material for: Perceptions and interest in applied educational neuroscience: a diagnostic study of Brazilian teachers
Source: Front Psychol. 2026 Aug 6;17:1895776. doi: 10.3389/fpsyg.2026.1895776 (PMC13489923; doi:10.3389/fpsyg.2026.1895776)
Supplement: Supplementary file 2 [file Data_Sheet_2.pdf]

Supp Table 1. Descriptive statistics for all Likert outcomes

Outcome	n	Mean	SD	Median	Q1	Q3
Memory and Learning	204	3.27	1.14	3.0	3.0	4.0
Child's Brain	204	2.78	1.28	3.0	2.0	4.0
ASD	204	2.85	1.30	3.0	2.0	4.0
Scientific Literacy/Critical Thinking	204	3.22	1.35	3.0	2.0	4.0
Dyslexia/Dysgraphia/Dyscalculia	204	2.51	1.39	3.0	1.0	3.0
Neurodevelopment/Brain Plasticity	204	2.55	1.48	3.0	2.0	4.0
ADHD	204	2.87	1.34	3.0	2.0	4.0
Learning Disorders	204	2.87	1.27	3.0	2.0	4.0
Emotions/Social-Emotional Learning	204	3.24	1.27	3.0	2.0	4.0
Adolescent Brain	204	2.47	1.34	2.5	2.0	3.0
Interest: ASD	204	3.79	1.32	4.0	3.0	5.0
Interest: ADHD	204	3.90	1.28	4.0	3.0	5.0
Interest: Dyslexia/Dysgraphia/Dyscalculia	204	3.66	1.36	4.0	3.0	5.0
Interest: Learning Disorders	204	3.98	1.27	4.0	3.0	5.0
Interest: Memory and Learning	204	4.16	1.19	5.0	3.8	5.0
Interest: Emotions/Social-Emotional Learning	204	4.08	1.14	5.0	3.0	5.0
Interest: Neurodevelopment/Brain Plasticity	204	3.96	1.37	5.0	3.0	5.0
Interest: Child's Brain	204	3.73	1.43	4.0	3.0	5.0
Interest: Adolescent Brain	204	3.65	1.48	4.0	2.0	5.0
Interest: Scientific Literacy/Critical Thinking	204	3.87	1.35	4.0	3.0	5.0
Asynchronous recorded classes	204	3.54	1.46	4.0	3.0	5.0
Synchronous remote classes	204	3.48	1.48	4.0	3.0	5.0
In-person classes	204	2.79	2.05	3.0	1.0	5.0
Individual reading	204	3.73	1.38	4.0	3.0	5.0
Synchronous remote workshops	204	3.44	1.53	4.0	3.0	5.0
In-person workshops	204	2.93	1.96	3.0	1.0	5.0
